# Supplementation with High Doses of Vitamin D to Subjects without Vitamin D Deficiency May Have Negative Effects: Pooled Data from Four Intervention Trials in Tromsø

**DOI:** 10.1155/2013/348705

**Published:** 2013-03-14

**Authors:** Rolf Jorde, Moira Strand Hutchinson, Marie Kjærgaard, Monica Sneve, Guri Grimnes

**Affiliations:** ^1^Tromsø Endocrine Research Group, Department of Clinical Medicine, University of Tromsø, 9037 Tromsø, Norway; ^2^Division of Internal Medicine, University Hospital of North Norway, 9038 Tromsø, Norway; ^3^Division of Surgery and Clinical Neuroscience, Department of Ophthalmology, Oslo University Hospital, 0424 Oslo, Norway

## Abstract

Data were pooled from four randomized clinical trials with vitamin D performed in Tromsø with weight reduction, insulin sensitivity, bone density, and depression scores as endpoints. Serum lipids, glycated hemoglobin (HbA_1c_), and high sensitivity C-Reactive Protein, (HS-CRP) were measured at baseline and after 6–12 months of supplementation with vitamin D 20 000 IU–40 000 IU per week versus placebo. A total of 928 subjects who completed the interventions were included. At baseline the mean serum 25-hydroxyvitamin D (25(OH)D) level in those given vitamin D was 55.9 (20.9) nmol/L and the mean increase was 82.4 (40.1) nmol/L. Compared with the placebo group there was in the vitamin D group at the end of the studies a slight, but significant, increase in HbA_1c_ of 0.04%, an increase in HS-CRP of 0.07 mg/L in those with serum 25(OH)D < 50 nmol/L, and in those with low baseline HDL-C and serum 25(OH)D < 50 nmol/L a slight decrease serum HDL-C of 0.08 mmol/L (*P* < 0.05). No serious side-effects were seen. In conclusion, in subjects without vitamin D deficiency, there is no improvement in serum lipids, HbA_1c_, or HS-CRP with high dose vitamin D supplementation. If anything, the effect is negative.

## 1. Introduction

In recent years there has been a great interest in the relation between vitamin D and health outcomes [1]. As the vitamin D receptor (VDR) is found in tissues throughout the body it is reasonable to assume that effects of vitamin D are not restricted to its classical effects on calcium metabolism [2]. Thus, the levels of serum 25-hydroxyvitamin D (25(OH)D), which is the metabolite used to evaluate a subject's vitamin D status [2], are in cross-sectional studies associated with risk factors for type 2 diabetes (T2DM) and cardiovascular disease [3–7] and in prospective studies associated with increased risk of these diseases and also cancer and even death [1, 8–11]. One would therefore expect that it should be easy to demonstrate a positive effect of vitamin D supplementation on health, but so far the evidence from properly performed randomized clinical trials (RCTs) is lacking.

We have previously performed four large RCTs with high dose vitamin D intervention in Tromsø, northern Norway, studying specifically the effects on weight [12], insulin sensitivity [13], bone density [14], and depression scores [15]. In addition to these endpoints we have also included other measures of glucose and lipid metabolism and inflammation markers. We have included more than 900 subjects in these studies, a number high enough to disclose an effect on these secondary endpoints, if present. Furthermore, it also enabled us to examine separately subjects with low serum 25(OH)D levels, subjects with deranged lipid and/or glucose metabolism, and also combinations of these subgroups. 

## 2. Materials and Methods

### 2.1. Patients

In the present study we have pooled data from four intervention studies.

- The vitamin D and obesity study in which 438 subjects 21–70 years old and with BMI 28–47 kg/m^2^ were included and randomized to 40 000 IU vitamin D per week, 20 000 IU vitamin D per week, or placebo for one year. All subjects were given 500 mg calcium daily [12]. In the present study those given 40 000 and 20 000 IU were combined to one vitamin D group. The study was registered at ClinicalTrials.gov (NCT00243256).
- Vitamin D and insulin sensitivity study where 108 subjects 30–75 years old with serum 25(OH)D < 50 nmol/L were included and randomized to vitamin D 20 000 IU twice per week versus placebo for six months [13]. The study was registered at ClinicalTrials.gov (NCT00809744).
- The vitamin D and bone density study including 297 postmenopausal women 50–80 years old, with a T-score in total hip or lumbar spine (L2-4) ≤ −2.0 and randomized to vitamin D 20 000 IU twice per week versus placebo for one year. In addition all subjects were given daily supplements with 1 g calcium and 800 IU vitamin D [14]. The study was registered at ClinicalTrials.gov (NTC00491920).
- The vitamin D and depression study comprising 243 subjects 30–75 years old, with serum 25(OH)D ≤ 55 nmol/L measured in the sixth Tromsø study and randomized to vitamin D 20 000 IU twice per week versus placebo for six months [15]. The study was registered at ClinicalTrials.gov (NCT00960232).

### 2.2. Measurements

Height and weight were measured wearing light clothing and no shoes. BMI was calculated as weight divided by height squared. Blood samples were drawn before and at the end of the intervention period, fasting in the obesity and insulin sensitivity study and non-fasting in the bone density and depression study. Serum calcium, PTH, total cholesterol (TC), triglycerides (TG), HDL cholesterol (HDL-C), LDL cholesterol (LDL-C), apolipoprotein A1 (Apo A1), apolipoprotein B (Apo B), glycated hemoglobin (HbA_1c_), high sensitivity C-reactive protein (HS-CRP), and serum 25(OH)D were measured as previously described [12–15]. 

### 2.3. Statistical Analyses

Distribution of the dependent variables was evaluated with skewness and kurtosis and visual inspection of histograms and found normal except for HS-CRP and delta (value at the end of study minus value at baseline) HS-CRP. Because of negative values log-transformation could not be performed and therefore comparisons between groups regarding HS-CRP were performed with the Mann-Whitney *U* test. For the other variables comparisons between groups were done with Student's *t*-test and also with a general linear model with gender, baseline age, and BMI as covariates. Interactions between treatment groups, gender, and use of lipid lowering medication were also tested in this model. The data are shown as mean (SD) except for HS-CRP which are shown as median (25, 75 percentile). A *P* value < 0.05 was considered statistically significant.

## 3. Results

In the obesity, insulin sensitivity, bone density, and depression studies a total number of 333, 94, 273, and 228 subjects, respectively, completed the intervention period and had complete data sets. At baseline there were no significant differences between the vitamin D and placebo groups except for age and BMI (Table 1). The subjects in the combined vitamin D group had significantly lower age than those in the placebo group (53.1 (11.6) versus 55.7 (10.8) years, *P* < 0.05)) and significantly higher BMI (29.7 (5.5) versus 28.6 (5.6) kg/m^2^, *P* < 0.05). The difference in age was mainly due to a significant difference between the vitamin D and placebo groups in the obesity study (48.1 (11.3) versus 51.4 (10.6) years, *P* < 0.05). 

### 3.1. Effects of Vitamin D Supplementation

In all the studies there was an increase in serum 25(OH)D and a decrease in serum PTH after vitamin D supplementation, as expected (Table 2). There was no significant difference in delta BMI neither between the groups pooled together, nor in the separate study groups, when only evaluating subjects with low serum 25(OH)D levels (< 50 nmol/L) or when stratifying according to baseline BMI (data not shown).

Among the 928 subjects, 70 were on lipid lowering medication and were therefore excluded when analyzing the effect on serum lipids. Furthermore, for HbA_1c_ there was a significant interaction between treatment groups and use of lipid lowering medication (*P* < 0.05), which was also borderline significant for HS-CRP (*P* = 0.07). In the following, the delta values for the lipids, HbA_1c_, and HS-CRP are therefore presented in the 858 subjects not using lipid lowering medication, and with the four groups pooled together unless otherwise stated. When pooling all the studies together, there were for the serum lipids no significant differences between the delta values for the vitamin D and the placebo groups. However, in the osteoporosis group a significant increase in serum HDL-C after vitamin D was seen when compared to placebo, but this difference was not statistically significant after adjusting for age, gender, and BMI. For HbA_1c_ there was in the combined vitamin D group an increase in HbA_1c_ of 0.02 (0.26)%, whereas in those given placebo there was a decrease of 0.02 (0.26)% (*P* < 0.05 between groups). This difference was also statistically significant after adjusting for age, gender, and BMI. For delta HS-CRP there was in the combined group a slight increase in those given vitamin D whereas in the placebo group there was a slight decrease, but the difference was not statistically significant (Table 2). 

### 3.2. Effect of Baseline 25(OH)D

To examine the effect of baseline serum 25(OH)D levels, separate analyses were made in subjects with serum 25(OH)D < 50 nmol/L (*n* = 377). This did not reveal any significant differences regarding delta values for the lipids. For delta HbA_1c_ the difference between those given vitamin D (*n* = 214) and placebo (*n* = 163) increased slightly (0.06 (0.25)% versus 0.01 (0.25)%, resp.), but it was no longer statistically significant. Also for delta HS-CRP the difference between those given vitamin D and placebo increased (0.04 (−0.43, 0.54) mg/L versus −0.04 (−0.89, 0.43) mg/L), and this difference was now statistically significant (*P* < 0.05). Further lowering the baseline 25(OH)D to < 40 nmol/L (*n* = 202) or < 30 nmol/L (*n* = 72) did not change the results substantially.

### 3.3. Effect of Baseline Lipid, HbA_1c_, and HS-CRP Levels on Effects of Vitamin D Supplementation

Separate analyses were performed for subjects with serum TC, TG, LDL-C, Apo B, and HbA_1c_ above the respective 75th percentiles and for subjects with serum HDL-C and Apo A1 below the 25th percentiles. For TC, TG, LDL-C, Apo B, HS-CRP, and HbA_1c_ subgroups no significant differences in delta values between those given vitamin D versus placebo were found. 

However, for HDL-C the effects of vitamin D supplementation appeared to depend on the baseline serum HDL-C and Apo A1 levels. Thus, for subjects with baseline HDL-C and Apo A1 below the 25th percentiles supplementation with vitamin D caused a significant (*P* < 0.05) decrease in serum HDL-C (but not in other lipids) as compared to placebo. For baseline HDL-C below the 25th percentile (<1.21 mmol/L) the delta HDL values were for the 144 subjects given vitamin D 0.00 (0.17) mmol/L versus 0.06 (0.16) mmol/L in the 96 subjects given placebo. Correspondingly, for subjects with baseline Apo A1 below the 25th percentile (<1.35 g/L) the delta HDL-C values were −0.01 (0.15) g/L in the 137 subjects given vitamin D and 0.04 (0.16) g/L in the 82 subjects given placebo. These differences were also statistically significant after adjustment for age, gender, and BMI. 

### 3.4. Combined Effects of Baseline Lipid, HbA_1c_, HS-CRP, and 25(OH)D Levels

Combinations of TC, TG, LDL, Apo B, HS-CRP 50th or 75th percentile subgroups, or Apo A1 50th or 25th subgroups with serum 25(OH)D < 50 nmol/L (or even lower cut-offs) did not reveal significant differences in delta values between those given vitamin D versus placebo.

However, for those with serum HDL-C below the 25th percentile (<1.21 mmol/L) and with serum 25(OH)D < 50 nmol/L, the difference in delta HDL-C between the vitamin D (*n* = 76) and placebo group (*n* = 58) was now even more pronounced (0.00 (0.11) mmol/L versus 0.08 (0.17) mmol/L, *P* < 0.001 after adjustment for age, gender and BMI). Similarly, for those with HbA_1c_ at baseline above the 75th percentile (>5.90%) and with serum 25(OH)D < 50 nmol/L, the difference in delta HbA_1c_ between those given vitamin D (*n* = 26) versus placebo (*n* = 30) was also larger (−0.04 (0.29)% and −0.19 (0.30)%, resp., *P* < 0.05 after adjustment for age, gender, and BMI).

## 4. Discussion

In the present study we have found high dose vitamin D supplementation to cause a slight but significant increase in HbA_1c_ and HS-CRP, and a decrease in serum HDL-C. Similarly, we have previously published the observation of a slight increase in systolic blood pressure by vitamin D supplementation in the obesity study [16]. 

The reason for starting the obesity, insulin sensitivity, bone density, and depression studies was the hypothesis that high doses of vitamin D (20 000–40 000 IU per week) would have beneficial effects even in a fairly vitamin D sufficient population as the one in Tromsø, northern Norway. However, in the original four publications [12–15] we did not see any clear benefits on the main endpoints body weight, insulin sensitivity as evaluated by a hyperglycemic clamp, bone density, or symptoms of depression. This could be related to power if the true effect of vitamin D supplementation is small, to inclusion of subjects with adequate vitamin D status where an effect of vitamin D supplementation would be hard to disclose, and to studying subjects with basically normal lipid and glucose metabolism and without severe symptoms of depression. We therefore pooled all the four studies together which at least to some extent enabled us to overcome the previous shortcomings. However, even with this approach we were not able to find any beneficial effect of high dose vitamin D supplementation. On the contrary, possible negative effects regarding lipid and glucose metabolism as well as on the inflammation marker HS-CRP were seen. These effects were small, with an absolute increase of 0.04% for mean HbA_1c_, a decrease in mean HDL-C of 0.05 mmol/L, and an increase in median HS-CRP of 0.05 mg/L in the vitamin D group versus the placebo group. It was also noteworthy that looking separately at subjects with lower serum 25(OH)D levels at baseline did not make high dose vitamin D supplementation appear more favorable as compared to subjects with higher baseline vitamin D levels. These effects individually, if true, can hardly be considered to be of clinical significance, at least not in the short run. However, if added together and if persisting over years, there might at a population level be negative effects. 

Our negative results are in line with recent reviews, meta-analyses, and large studies regarding serum lipids and vitamin D supplementation [16–18], and we have previously concluded in an editorial that it is questionable if more such studies are needed [19]. With a few exceptions [20] most intervention studies have not been able to show an effect on glucose metabolism [21, 22], and similarly, most studies on vitamin D effects on inflammatory markers have also been negative [22–24].

The present study does have many shortcomings. First of all, we have pooled data from different studies together, with length of intervention ranging from six months to one year, with doses of vitamin D ranging from 20 000 IU per week up to 45 600 IU per week; the subjects included were highly selected based on BMI, BMD, and serum 25(OH)D levels; in two of the studies the placebo group and the vitamin D group were given calcium; and in one of the studies the high dose of vitamin D (45 600 IU per week) was compared with a standard dose of 5600 IU per week. Accordingly, we cannot firmly state that high doses of vitamin D are harmful, but it is fair to conclude that it is highly unlikely that high doses of vitamin D will have a significant positive effect on serum lipids or measures of glucose metabolism. On the other hand, one may also interpret our data the other way around that giving these high doses of vitamin D appears to be safe. 

Our study also has some strengths. We pooled data from studies from a single centre, and to our knowledge there are no RCTs that have included as many subjects as we have. Also, we gave high doses of vitamin D with the expected effect on the serum 25(OH)D levels. 

It must also be emphasized that we have only measured risk markers, and what is of real importance is the effect of vitamin D on hard endpoints like development of T2DM, cardiovascular disease, and ultimately mortality. So far this has not been evaluated in studies designed for that purpose, and meta-analyses of intervention studies (primarily with fractures or BMD as main endpoints) have been inconclusive [25, 26]. There are several large RCTs ongoing in Europe, New Zealand, and the USA [27] that will answer these questions in populations with a reasonable good vitamin D status. However, it should be recalled that vitamin D deficiency is a worldwide problem [28], and RCTs are particularly important in populations at higher risk than those at present being studied [29]. 

## 5. Conclusion

We have not found a beneficial effect of high dose vitamin D supplementation on lipid and glucose metabolism in a population that is not vitamin D deficient. If anything, the effect appears to be negative.

## Figures and Tables

**Table 1:** Baseline characteristics in all subjects, the four separate studies, and according to intervention.

	All subjects	Obesity study	Insulin sensitivity study	Bone density study	Depression study	Intervention, pooled data	
						Vitamin D	Placebo
*N*	928	333	94	273	228	523	405
Men (%)	29.9	38.4	52.1	0	43.9	32.1	27.2
Age (years)	54.2 (11.3)	49.2 (11.2)	52.1 (9.2)	63.1 (7.2)	51.8 (10.2)	53.1 (11.6)*	55.7 (10.8)
Using lipid medication (%)	7.3	3.9	5.3	11.4	8.8	5.9	9.4
BMI (kg/m^2^)	29.2 (5.6)	34.6 (3.9)	26.8 (3.0)	24.7 (3.3)	27.7 (4.1)	29.7 (5.5)*	28.6 (5.6)
Serum 25(OH)D (nmol/L)	55.9 (20.9)	54.1 (16.8)	40.8 (13.1)	71.0 (22.6)	47.5 (15.6)	55.9 (20.6)	56.4 (21.3)
Serum calcium (mmol/L)	2.32 (0.09)	2.31 (0.10)	2.31 (0.09)	2.36 (0.08)	2.28 (0.08)	2.32 (0.10)	2.32 (0.09)
Serum PTH (pmol/L)	5.17 (1.72)	5.41 (1.71)	5.20 (1.52)	5.05 (1.65)	4.98 (1.87)	5.16 (1.65)	5.20 (1.82)
Serum TC (mmol/L)	5.56 (1.00)	5.41 (1.00)	5.57 (1.07)	5.65 (0.92)	5.64 (1.05)	5.56 (1.01)	5.55 (0.99)
Serum TG (mmol/L)	1.40 (0.84)	1.57 (0.93)	1.25 (0.85)	1.11 (0.51)	1.59 (0.91)	1.43 (0.89)	1.37 (0.79)
Serum HDL-C (mmol/L)	1.57 (0.48)	1.40 (0.37)	1.50 (0.40)	1.88 (0.48)	1.47 (0.46)	1.55 (0.46)	1.59 (0.50)
Serum LDL-C (mmol/L)	3.66 (0.94)	3.86 (0.95)	3.51 (0.99)	3.53 (0.85)	3.57 (0.95)	3.69 (0.94)	3.61 (0.94)
Serum Apo A1 (g/L)	1.56 (0.29)	1.43 (0.25)	1.52 (0.28)	1.75 (0.26)	1.55 (0.29)	1.55 (0.29)	1.57 (0.29)
Serum Apo B (g/L)	0.98 (0.24)	1.03 (0.23)	0.95 (0.26)	0.96 (0.22)	0.97 (0.25)	0.99 (0.24)	0.98 (0.24)
HbA_1c_ (%)	5.65 (0.35)	5.65 (0.36)	5.56 (0.36)	5.71 (0.34)	5.64 (0.36)	5.65 (0.36)	5.67 (0.35)
Serum HS-CRP (mg/L)	1.45 (0.77, 3.22)	2.47 (1.34, 4.51)	1.10 (0.59, 2.55)	1.02 (0.50, 2.01)	1.28 (0.77, 2.96)	1.51 (0.77, 3.35)	1.42 (0.78, 3.16)

Data are shown as mean (SD) or median (25, 75 percentile). BMI: body mass index; 25(OH)D: 25-hydroxyvitamin D; PTH: parathyroid hormone; TC: total cholesterol; TG: triglycerides; HDL-C: HDL cholesterol; LDL-C: LDL cholesterol; Apo A1: apolipoprotein A1; Apo B: apolipoprotein B; HbA_1c_: glycated hemoglobin; HS-CRP: high sensitivity C-reactive protein.

*P < 0.05 versus placebo group; Student's *t*-test.

**Table 2:** Delta values (Δ, value at the end of study minus value at baseline) in the four separate studies and pooled together.

	Obesity study		Insulin sensitivity study		Bone density study		Depression study		All studies together	
	Vitamin D	Placebo	Vitamin D	Placebo	Vitamin D	Placebo	Vitamin D	Placebo	Vitamin D	Placebo
All subjects (*N*)	221	112	49	45	134	139	119	109	523	405
ΔBMI (kg/m^2^)	0.0 (1.2)	0.2 (1.4)	−0.3 (0.9)	−0.1 (1.0)	−0.0 (1.0)	−0.1 (2.1)	0.1 (0.8)	0.1 (0.8)	0.0 (1.0)	0.0 (1.5)
ΔSerum 25(OH)D nmol/L	49.3 (21.7)***	−2.2 (9.5)	100.5 (27.8)***	3.7 (17.5)	114.7 (34.5)***	17.9 (18.8)	100.2 (29.6)***	4.7 (14.2)	82.4 (40.1)***	7.2 (17.3)
ΔSerum calcium (mmol/L)	−0.01 (0.12)	−0.01 (0.11)	−0.06 (0.08)	−0.07 (0.09)	0.02 (0.09)	−0.01 (0.10)	0.02 (0.08)*	−0.01 (0.08)	0.00 (0.10)*	−0.01 (0.10)
ΔSerum PTH (pmol/L)	−0.86 (1.47)**	−0.28 (1.58)	−0.16 (1.42)*	0.67 (1.90)	−1.19 (1.38)***	−0.62 (1.52)	−0.78 (1.25)***	0.19 (1.36)	−0.86 (1.42)***	−0.16 (1.60)
Subjects not on lipid medication (*N*)	214	105	47	42	119	123	112	96	492	366
ΔSerum TC (mmol/L)	−0.12 (0.58)	−0.19 (0.54)	0.01 (0.58)	−0.05 (0.64)	0.18 (0.74)	0.06 (0.78)	−0.12 (0.53)	−0.07 (0.70)	−0.04 (0.62)	−0.06 (0.68)
ΔSerum TG (mmol/L)	0.01 (0.89)	0.07 (0.59)	−0.04 (0.67)	−0.02 (0.81)	0.07 (0.58)	0.02 (0.47)	−0.16 (0.73)	−0.11 (0.62)	−0.02 (0.77)	0.00 (0.59)
ΔSerum HDL-C (mmol/L)	−0.08 (0.17)	−0.09 (0.18)	0.02 (0.19)	−0.01 (0.25)	0.03 (0.25)	−0.04 (0.27)	0.03 (0.17)	0.08 (0.19)	−0.02 (0.20)	−0.02 (0.23)
ΔSerum LDL-C (mmol/L)	−0.26 (0.51)	−0.33 (0.48)	0.07 (0.47)	0.07 (0.54)	−0.02 (0.59)	−0.08 (0.66)	−0.04 (0.47)	−0.01 (0.61)	−0.12 (0.53)	−0.12 (0.60)
ΔSerum Apo A1 (g/L)	0.01 (0.17)	0.00 (0.16)	−0.03 (0.23)	−0.03 (0.21)	0.06 (0.17)	0.04 (0.19)	−0.09 (0.15)	−0.06 (0.16)	−0.01 (0.18)	−0.01 (0.18)
ΔSerum Apo B (g/L)	−0.01 (0.13)	−0.02 (0.13)	−0.06 (0.10)	−0.05 (0.12)	0.01 (0.15)	0.00 (0.15)	−0.04 (0.11)	−0.03 (0.13)	−0.01 (0.13)	−0.02 (0.14)
ΔHbA_1c_ (%)	0.10 (0.22)	0.07 (0.23)	0.12 (0.32)	0.05 (0.29)	−0.11 (0.28)	−0.12 (0.27)	−0.03 (0.20)	−0.01 (0.22)	0.02* (0.26)	−0.02 (0.26)
ΔSerum HS-CRP (mg/L)	0.11 (−0.60, 1.00)	−0.10 (−0.69, 0.88)	−0.04 (−0.54, 0.23)	−0.02 (−0.20, 0.49)	0.02 (−0.22, 0.43)	0.00 (−0.53, 0.31)	−0.06 (−0.41, 0.35)	−0.01 (−0.85, 0.39)	0.02 (−0.47, 0.59)	−0.03 (−0.55, 0.45)

*P < 0.05, **P < 0.01, and ***P < 0.001 versus placebo group; general linear model adjusted for age, BMI, and gender. Data are shown as mean (SD) or median (25, 75 percentile). BMI: body mass index; 25(OH)D: 25-hydroxyvitamin D; PTH: parathyroid hormone; TC: total cholesterol; TG: triglycerides; HDL-C: HDL cholesterol; LDL-C: LDL cholesterol; Apo A1: apolipoprotein A1; Apo B: apolipoprotein B; HbA_1c_: glycated hemoglobin; HS-CRP: high sensitivity C-reactive protein.
